# Inactivation of *SDH* and *FH* cause loss of 5hmC and increased H3K9me3 in paraganglioma/pheochromocytoma and smooth muscle tumors

**DOI:** 10.18632/oncotarget.6091

**Published:** 2015-10-12

**Authors:** Attje S. Hoekstra, Marieke A. de Graaff, Inge H. Briaire-de Bruijn, Cor Ras, Reza Maleki Seifar, Ivonne van Minderhout, Cees J. Cornelisse, Pancras C.W. Hogendoorn, Martijn H. Breuning, Johnny Suijker, Esther Korpershoek, Henricus P.M. Kunst, Norma Frizzell, Peter Devilee, Jean-Pierre Bayley, Judith V.M.G. Bovée

**Affiliations:** ^1^ Department of Human Genetics, Leiden University Medical Center, Leiden, The Netherlands; ^2^ Department of Pathology, Leiden University Medical Center, Leiden, The Netherlands; ^3^ Department of Biotechnology, Delft University of Technology, Delft, The Netherlands; ^4^ Department of Clinical Genetics, Leiden University Medical Center, Leiden, The Netherlands; ^5^ Department of Pathology, Josephine Nefkens Institute, Erasmus Medical Center Rotterdam, Rotterdam, The Netherlands; ^6^ Department of Otorhinolaryngology, Head and Neck Surgery, Radboud University Nijmegen Medical Center, Nijmegen, The Netherlands; ^7^ Department of Pharmacology, Physiology & Neuroscience, School of Medicine, University of South Carolina, Columbia, SC, USA

**Keywords:** paraganglioma, succinate dehydrogenase, fumarate hydratase, hereditary leiomyomatosis and renal cell carcinoma, methylation

## Abstract

Succinate dehydrogenase (SDH) and fumarate hydratase (FH) are tricarboxylic acid (TCA) cycle enzymes and tumor suppressors. Loss-of-function mutations give rise to hereditary paragangliomas/pheochromocytomas and hereditary leiomyomatosis and renal cell carcinoma. Inactivation of SDH and FH results in an abnormal accumulation of their substrates succinate and fumarate, leading to inhibition of numerous α-ketoglutarate dependent dioxygenases, including histone demethylases and the ten-eleven-translocation (TET) family of 5-methylcytosine (5mC) hydroxylases. To evaluate the distribution of DNA and histone methylation, we used immunohistochemistry to analyze the expression of 5mC, 5-hydroxymethylcytosine (5hmC), TET1, H3K4me3, H3K9me3, and H3K27me3 on tissue microarrays containing paragangliomas/pheochromocytomas (*n* = 134) and hereditary and sporadic smooth muscle tumors (*n* = 56) in comparison to their normal counterparts. Our results demonstrate distinct loss of 5hmC in tumor cells in *SDH-* and *FH*-deficient tumors. Loss of 5hmC in *SDH*-deficient tumors was associated with nuclear exclusion of TET1, a known regulator of 5hmC levels. Moreover, increased methylation of H3K9me3 occurred predominantly in the chief cell component of *SDH* mutant tumors, while no changes were seen in H3K4me3 and H3K27me3, data supported by *in vitro* knockdown of *SDH* genes. We also show for the first time that *FH*-deficient smooth muscle tumors exhibit increased H3K9me3 methylation compared to wildtype tumors. Our findings reveal broadly similar patterns of epigenetic deregulation in both *FH*- and *SDH*-deficient tumors, suggesting that defects in genes of the TCA cycle result in common mechanisms of inhibition of histone and DNA demethylases.

## INTRODUCTION

The tricarboxylic acid (TCA) cycle enzymes isocitrate dehydrogenase (IDH), succinate dehydrogenase (SDH) and fumarate hydratase (FH) are mutated in a subset of human cancers, leading to alterations in cell metabolism. In the TCA cycle, the SDH complex converts succinate to fumarate, while FH catalyzes the hydroxylation of fumarate to L-malate. Germline mutations in *SDHA* [1], *SDHB* [2], *SDHC* [3], *SDHD* [4], and *SDHAF2* [5] cause paraganglioma/pheochromocytoma (PGL/PCC). PGL of the head and neck arise most commonly in the carotid body, a chemoreceptor organ with two predominant cell types: the chief (type I) cells, which represent the neoplastic population in paragangliomas [6], and the surrounding supportive sustentacular (type II) cells. Inactivating germline mutations of *FH* result in hereditary leiomyomatosis and renal cell carcinoma (HLRCC), which is inherited in an autosomal dominant manner [7, 8]. Leiomyomas, benign smooth muscle tumors predominantly found in the skin and uterus, are the most common tumor type in HLRCC, but papillary type 2 renal cell carcinomas may also occur, although less frequently. Rare germline mutations in *FH* were recently reported in patients with PGL/PCC [9-11].

Although the mechanisms by which mutations in metabolic enzymes promote tumor formation are still poorly understood, the stabilization of hypoxia inducible factor (HIF) under conditions of normoxia is the most widely studied mechanism. Inactivation of SDH and FH leads to accumulation of the respective substrates succinate and fumarate, which inhibit α-ketoglutarate (α-KG) dependent HIF prolyl hydroxylases, leading to HIF activation [12, 13]. Other dioxygenases, including histone demethylases and the TET (ten-eleven translocation) family of 5-methylcytosine (5mC) hydroxylases, are also inhibited by succinate and fumarate accumulation [14-16]. The JmjC domain-containing histone demethylases and the TET family of DNA hydroxylases play central roles in epigenetic control of genomic information. While the JmjC domain-containing histone demethylases catalyze the oxidation of methyl groups on the lysine residues of histones H3 and H4 [17], TET1 and TET2 are responsible for the oxidation of 5mC to 5-hydroxymethylcytosine (5hmC), a process requiring α-KG and oxygen [18]. TET3 is mainly involved in the oxidation of 5mC to 5hmC in zygotic paternal DNA after fertilization [19].

Mutations in *SDH* are also found in gastrointestinal stromal tumors, in addition to the more commonly occurring mutations in *KIT* or *PDGFRA*. In these tumors, *SDH* mutations were shown to be associated with global hypermethylation and loss of 5hmC [20]. Furthermore, a PGL/PCC cohort showed a hypermethylation phenotype in *SDH* mutant tumors, reminiscent of the methylation signature of gliomas with *IDH* mutations [11, 20]. Enchondromas carrying an *IDH1* mutation also display a hypermethylation profile [21]. The common *IDH1* and *IDH2* mutations cause a gain-of-function and confer a neomorphic catalytic activity that allows the synthesis and accumulation of the oncometabolite 2-hydroxyglutaric acid (2HG). Due to the structural similarity to α-KG, 2HG competitively inhibits α-KG-dependent TET and histone demethylase enzyme families [22]. Intriguingly, a study by Letouzé *et al.* [11] included a hypermethylated PGL/PCC subgroup in which the only tumor sample without *SDH* mutations was shown to harbor germline inactivating *FH* mutations. A study in five patients with *FH*-deficient PGL/PCC reported loss of hydroxylation of 5mC in tumor cells [9]. This was also seen in *SDH*-deficient PGL/PCC and *IDH* mutant gliomas, suggesting that a common pathophysiological mechanism leads to alterations in DNA methylation. Furthermore, increased expression of the repressive trimethylation of H3K9 (H3K9me3) and a trend towards an increase in trimethylation of H3K27 (H3K27me3) was reported in *IDH1* mutant gliomas, while no differences were observed in the active trimethylation of H3K4 [23]. To date, DNA and histone methylation profiles have not been reported for *FH*-deficient smooth muscle tumors. In addition, the role of TET1 has not yet been explored in *SDH* mutant PGL/PCC or in *FH*-deficient tumors.

Using immunohistochemistry, we investigated the distribution of 5mC, 5hmC, TET1, and histone methylation in *SDH*- mutant PGL/PCC and in *FH*-deficient smooth muscle tumors in comparison to non-S*DH* or FH-mutated PGL/PCC and smooth muscle tumors, respectively. Interestingly, we found a similar pattern of epigenetic deregulation in *FH*-deficient smooth muscle tumors compared to *SDH*-deficient PGL/PCC, both in terms of loss of 5hmC expression and increased trimethylation of H3K9 in tumor cells.

## RESULTS

### Low prevalence of FH mutations in smooth muscle tumors and absence in PGL

To estimate the prevalence of *FH* mutations in smooth muscle tumors and to exclude *FH* mutations in *SDH* wildtype PGL, we performed immunohistochemistry for 2-succinocysteine (2SC), a robust biomarker for *FH* mutations [9, 24-26]. Of all hereditary and sporadic smooth muscle tumors (*n* = 56), 1 uterine LM and 1 cutaneous LM from a patient with suspected HLRCC, and 2 LMS tumors were positive for 2SC, indicating the presence of an *FH* mutation (Table 1, Supplemental Figure 2A, 2B). Of the LM/leiomyosarcoma (LMS) tumors with an unknown *FH* mutation status, 1 uterine LM and 1 cutaneous LM (from a patient with suspected HLRCC), and 2 LMS tumors were positive for 2SC (Table 1, Supplemental Figure 2A, 2B). All PGL/PCC tumors were negative for 2SC. In addition, negative SDHB staining and positive SDHA staining correlated exactly with the known mutation status in all *SDHB, SDHC, SDHD* and *SDHAF2* mutant tumors (Supplemental Figure 2C, 2D).

**Table 1:** Genomic characteristics of tumor specimens

Mutation status	Paraganglioma (n=109)	Pheochromocytoma (n=25)	Uterine Leiomyoma (n=9)	Cutaneous Leiomyoma (n=3)	Leiomyosarcoma (n=44)
SDHB	2				
SDHC	1				
SDHD	59	1			
SDHAF2	12				
VHL		9			
NF1		2			
MEN1		1			
RET		3			
Nonfamilial^1^	35	9	7	1	42
FH	0	0	2	2	2*

Mutation status is indicated for each tumor sample, in absolute numbers of each group size. Abbreviations: SDH, succinate dehydrogenase; VHL, von hippel lindau; NF1, neurofibromatosis type 1; MEN1, multiple endocrine neoplasia type 1; RET, rearranged during transfection (mutation gives rise to multiple endocrine neoplasia type 2); FH, fumarate hydratase.

1 No SDHA/B/C/D/AF2/VHL or FH mutation.

* FH mutation detected by 2SC staining.

### Loss of 5hmC in SDH and FH mutant tumors

**Figure 1:**
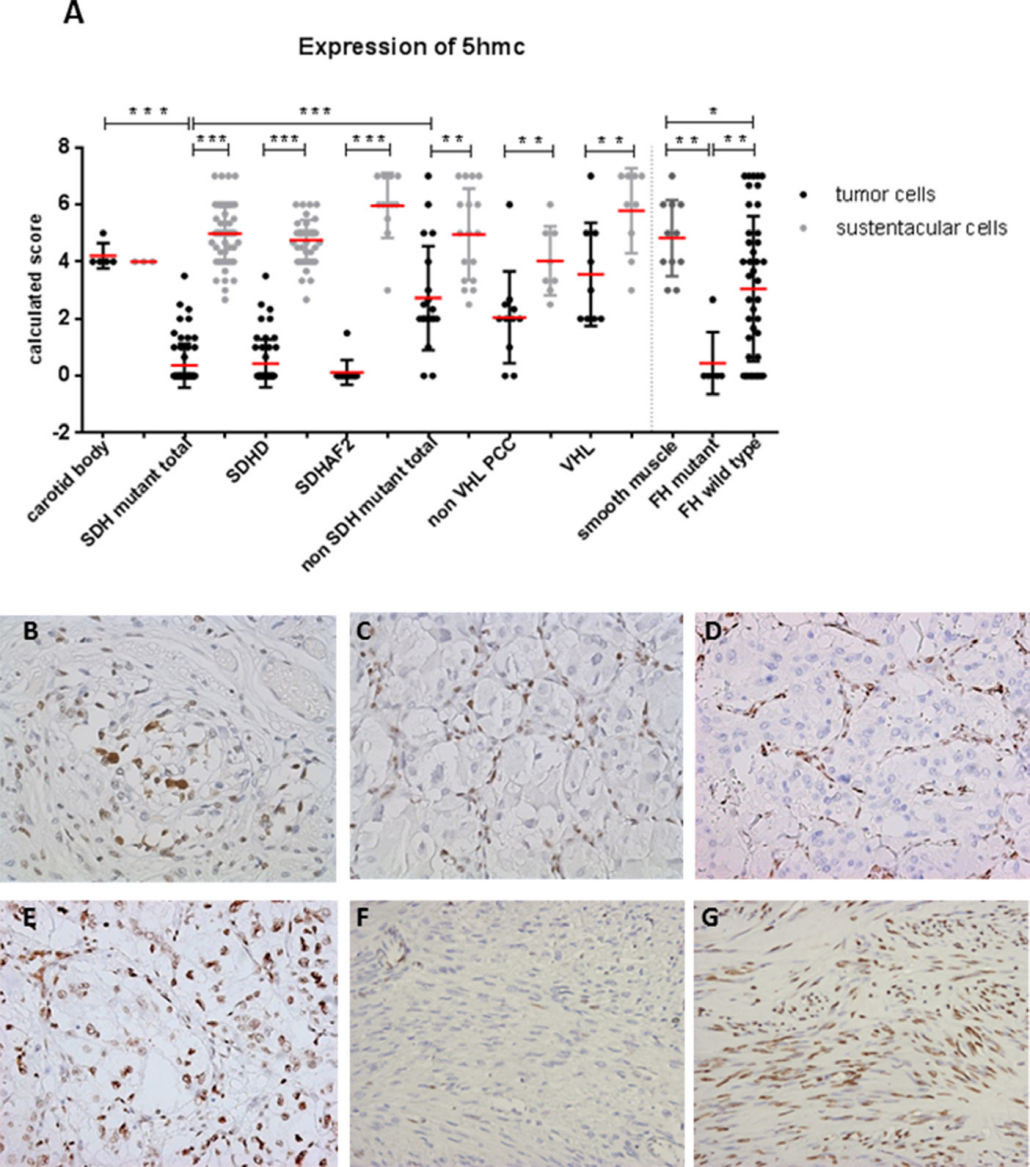
Loss of 5hmC expression in tumor cells of SDH and FH mutant tumors **A.** Dot plot presenting results of immunohistochemical 5hmC expression in tissues. Data are represented as calculated mean score ± standard deviation. *p < 0.05; **p < 0.001; ***p < 0.0001. **B.** Micrographs of representative staining (40x magnification) show strong immunostaining of sustentacular cell nuclei in normal carotid body and in **C.**-**E.** all *SDH-*related tumor types, whereas tumor cell staining (chief cell) was weaker or absent in *SDH*x-mutated tumors compared to **E.** *VHL* mutant PCC. **F.** Loss of 5hmC in tumor cells of *FH* mutant compared to **G.** *FH* wildtype.

**Figure 2:**
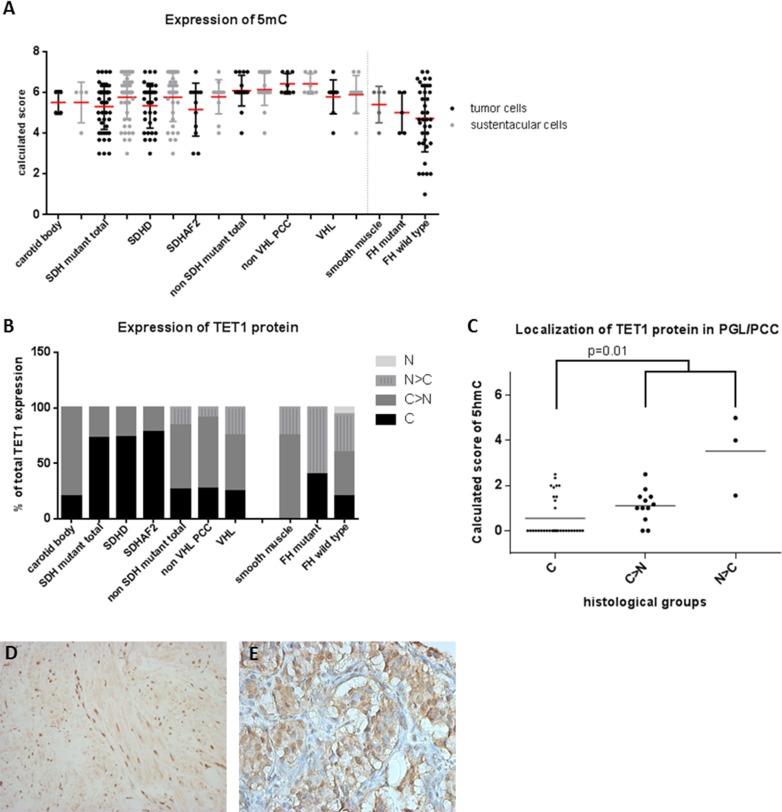
5mC and TET1 protein expression in SDH and FH mutant tumors **A.** Dot plot presenting results of immunohistochemical 5mC expression demonstrating a high expression in all tumors. Data are represented as calculated mean score ± standard deviation. **B.** TET1 expression and its subcellular localization, demonstrating nuclear exclusion in *SDH*x related tumors **C.** which is associated with low 5hmC expression levels (*p* = 0.01). **D.** Micrographs of representative staining (40x magnification) of TET1 are shown for *FH* mutant LM with predominantly nuclear staining and **E.** *SDHD* mutant tumor with cytoplasmic staining. Subcellular localization of the protein: C, exclusively cytoplasmic; C > N, predominantly cytoplasmic; N, exclusively nuclear; N > C, predominantly nuclear.

Since elevated intracellular succinate and fumarate competitively inhibit TET-catalyzed oxidation of 5mC to 5hmC [16], we analyzed expression of 5hmC and 5mC in *SDH/FH*-deficient and non-*SDH/FH* mutant tumors. In PGL/PCC, the expression of 5hmC differed markedly between chief cells and sustentacular cells within the same tumor (Figure 1A, 1C, 1D). Of the *SDH* mutant PGLs, 95% showed no or low expression (score 0-2) of 5hmC in chief cells, whereas 90% of the sustentacular cells in the same tumors showed high expression levels (score 4-7). When compared with chief cells in normal carotid bodies (Figure 1A, 1B), the chief cells in the tumor showed significantly lower expression of 5hmC (*p* = 0.0001). Similarly, 5hmC was significantly lower or even absent in *FH*-deficient tumors compared to normal smooth muscle tissue and *FH* wildtype tumors (Figure 1A, 1F-1G) (*p* = 0.0001). In PCC, the difference between tumor cells and sustentacular cells was less pronounced, but significant (*p* = 0.001) (Figure 1E). Likewise, the ratio of 5hmC expression in the chief cells and the sustentacular cells per tumor sample differed significantly between *SDH*-deficient tumors, normal carotid bodies, and non-*SDH* mutant tumors (Supplemental Fig. 3). We also analyzed 5mC, which is present at up to 40-fold greater levels in cells than 5hmC [27]. Perhaps unsurprisingly, the large shifts seen in the small 5hmC pool were not reflected in a detectable shift in the far larger 5mC pool, and 5mC was found to be highly expressed in all *SDH*- and *FH*-deficient tumors and controls (Figure 2A, 2D).

### Nuclear exclusion of TET1 is associated with loss of 5hmC in SDH mutant tumors

Since TET1 is responsible for the oxidation of 5mC to 5hmC and gliomas with loss of 5hmC expression have been reported to show nuclear exclusion of TET1 expression [28], we investigated this correlation in *SDH* and *FH* mutant tumors. Indeed, absence of nuclear staining for TET1 was more common in *SDH* mutant PGL (38 of 52, 73%) compared to either non-*SDH* mutated PGL/PCC (5/19, 26%) (*p* = 0.002) or normal carotid bodies (1/5, 20%) (*p* = 0.03) (Figure 2B, 2E). *SDH*-deficient tumors with cytoplasmic TET1 expression more frequently showed loss of 5hmC (Figure 2C, *p* = 0.01). Absence of nuclear staining was also more frequent in *FH*-deficient smooth muscle tumors (2 of 5, 40%), compared to *FH* wildtype tumors (9/45, 20%) or smooth muscle tissue (0/8). However, this difference was not significant (*p* = 0.5) and was not correlated to loss of 5hmC expression (*p* = 1.0).

### Increased H3K9me3 in SDH and FH mutant tumors

**Figure 3:**
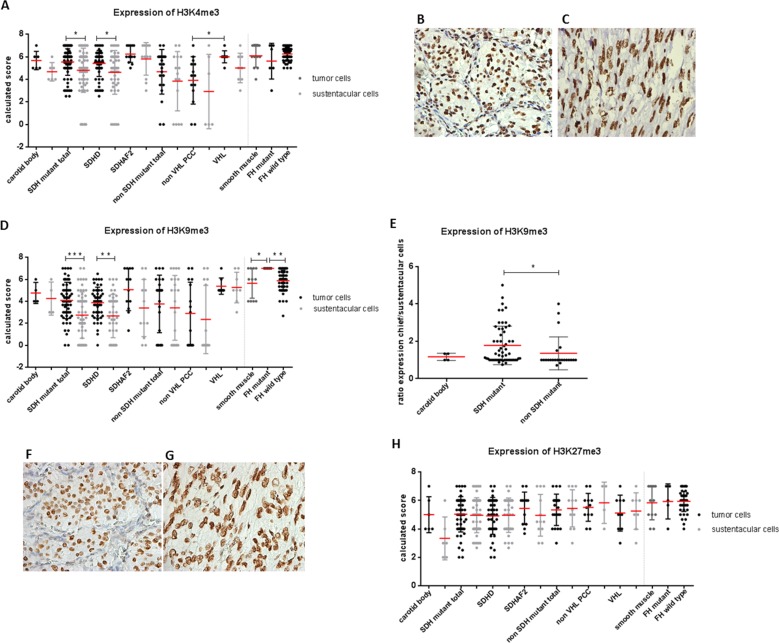
Expression of histone markers in SDH and FH mutant tumors **A.** Dot plot presenting results of immunohistochemical H3K4me3 levels in *SDH*x and *FH* mutant tumors. Micrographs of representative staining (40x magnification) are shown for **B.** *SDHD* mutant and **C.** *FH* mutant tumors. **D.** H3K9me3 expression levels are significantly increased in chief cells compared to sustentacular cells in *SDH* mutant tumors and in tumor cells of *FH*-deficient tumors compared to smooth muscle cells and *FH* wildtype tumors. **E.** Increased ratio of H3K9me3 expression observed in *SDH* mutant compared to non-*SDH* mutant tumors. Micrographs of representative staining (40x magnification) are shown for **F.** *SDHD* mutant and **G.** *FH* mutant tumors. **H.** No differences observed in H3K27me3 expression. Data are represented as calculated mean score ± standard deviation. *p < 0.05; **p < 0.001; ***p < 0.0001.

Succinate and fumarate have been shown to directly inhibit α-KG dependent histone demethylase activity in a manner similar to 2HG, resulting in increased methylation of various lysine residues of histone H3 [16, 23]. We therefore evaluated H3K4me3, H3K9me3, and H3K27me3 expression in *SDH* and *FH* mutant tumors by immunohistochemistry. Expression of trimethylated H3K4 was significantly increased in chief cells of *SDH* mutant tumors compared to sustentacular cells (Figure 3A, 3B) (*p* = 0.01). However, expression levels of H3K4me3 were also high in chief cells of normal carotid bodies and in non-*SDH* mutant tumors. No differences were seen in H3K4me3 expression between smooth muscle cells, *FH*-deficient, and *FH* wildtype tumors (Figure 3A, 3C).

The expression of H3K9me3 was significantly increased in chief cells compared to sustentacular cells in *SDH*-deficient tumors (*p* = 0.0001), but again, this was not significantly different from the expression in normal carotid bodies or non-*SDH* mutant tumors (Figure 3D, 3F). However, when we plotted the ratio of H3K9me3 expression per tumor sample by dividing the expression in the chief cells by the expression in the sustentacular cells within the same tumor, thus co-opting sustentacular cells as an inter-tumor control, expression levels of H3K9me3 were significantly increased in *SDH*-deficient tumors compared to non-*SDH* mutant tumors (Figure 3E) (*p* = 0.01). Regarding PCCs, H3K4me3 and H3K9me3 levels were increased in *VHL*-deficient tumors compared to non *VHL* mutant PCC. Interestingly, *FH*-deficient smooth muscle tumors showed significantly elevated levels of H3K9me3 compared to smooth muscle cells (*p* = 0.027) and *FH* wildtype tumors (Figure 3D, 3G) (*p* = 0.004). In contrast, no expression differences were seen for H3K27me3, with high expression in all tumors and controls, regardless of the manner of analysis (Figure 3H).

### Increased H3K9me3 upon inhibition of SDHD, SDHB or SDHAF2 *in vitro*

**Figure 4:**
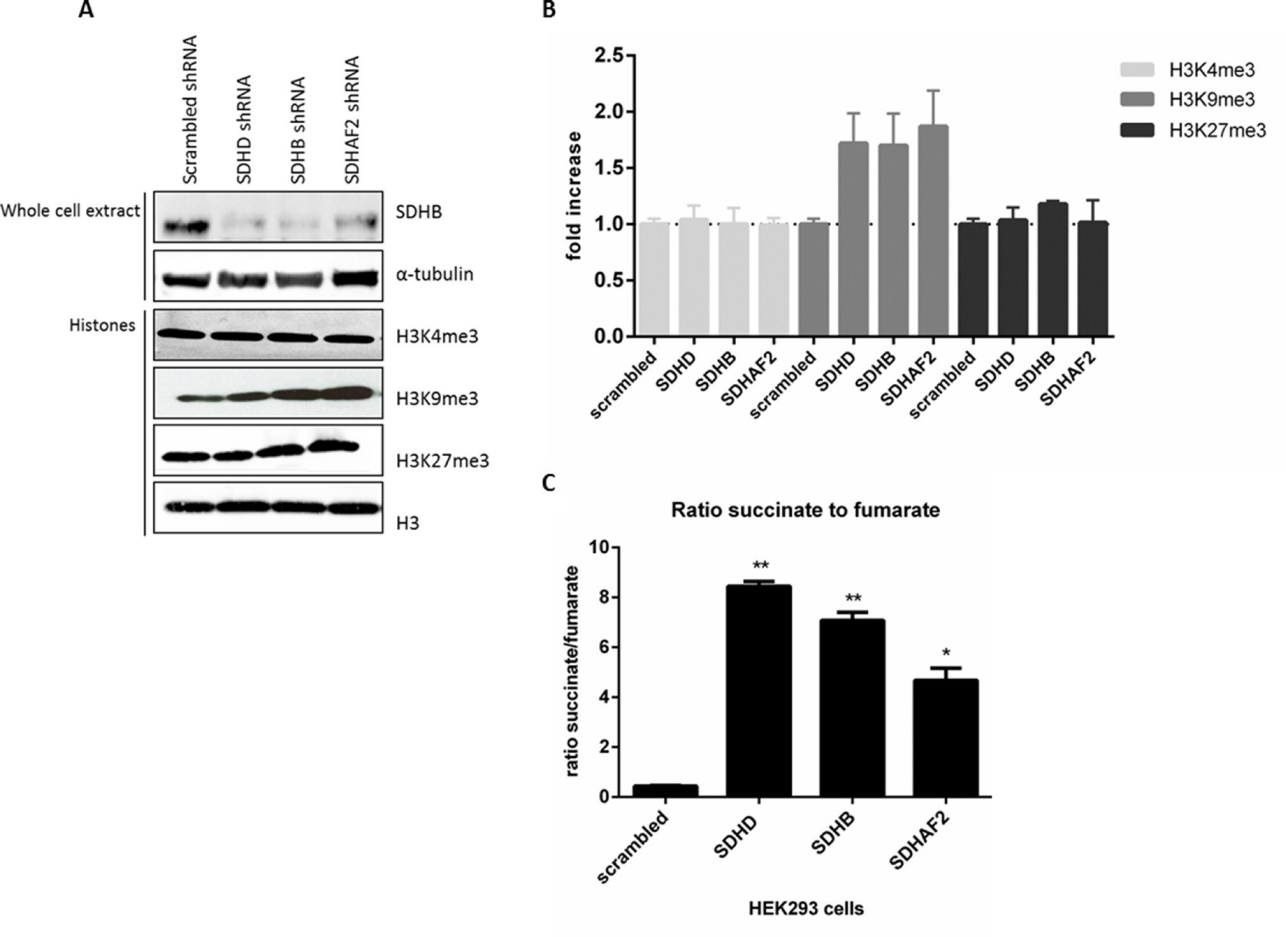
Increased H3K9me3 protein by succinate dehydrogenase gene inhibition **A.** HEK293 cells with stable knockdown of *SDHD, SDHB* and *SDHAF2* demonstrate decreased SDHB protein levels in total protein extract. α-Tubulin was used as a loading control. Histone lysine methylation levels were assessed in total histone fractions by western blotting with specific antibodies. Total H3 was used as a loading control. **B.** Quantification of western blotting demonstrates only H3K9me3 levels were increased by silencing of *SDHD* (by 1.72-fold), *SDHB* (by 1.7-fold) and *SDHAF2* (by 1.9-fold) in HEK293 cells compared to scrambled shRNA. **C.** HEK293 cells with stable knockdown of *SDHD, SDHB* and *SDHAF2* demonstrate a significant increased ratio of succinate to fumarate compared to scrambled cells, measured by LC-MS/MS. *p < 0.05; **p < 0.001.

In order to establish a direct causal link between histone methylation and loss of SDH, we derived subclones of HEK293 cells with stable knockdown of *SDHD, SDHB* and *SDHAF2*. Stable knockdown was confirmed by RT-PCR analysis of RNA expression levels and by immunoblotting, with decreased SDHB protein levels under all three conditions taken as a marker for *SDH* deficiency [29] (Figure 4A). Analysis of nuclear histones in these subclones revealed an increase in steady-state levels of H3K9me3 upon silencing of *SDHD* (by 1.7-fold), *SDHB* (by 1.7-fold), and *SDHAF2* (by 1.9-fold) (Figure 4B). Furthermore, silencing of *SDHD, SDHB,* and *SDHAF2* did not lead to increased trimethylation of H3K4 or H3K27 in HEK293 cells. To further validate the level of *SDHD, SDHB* and *SDHAF2* silencing in HEK293 cells, succinate and fumarate levels were quantified by LC-MS/MS. Succinate levels were increased in cells with knockdown of *SDHD, SDHB* or *SDHAF2* compared to scrambled cells. Likewise, the succinate-to-fumarate ratio was increased in the silenced cells compared to scrambled cells (Figure 4C), results which accord with those reported by Lendvai *et al*. [30].

## DISCUSSION

Loss-of-function mutations in *SDH* and *FH* leading to the accumulation of succinate and fumarate indirectly act as inhibitors of α-KG dependent dioxygenases. Here, we demonstrate that *SDH* and *FH* mutations can inhibit DNA and histone demethylases, leading to loss of 5hmC and increased H3K9me3 levels. We convincingly showed loss of 5hmC in chief cells in almost all (95%) *SDH*-deficient PGL/PCC. Moreover, *FH*-deficient smooth muscle tumors (83%) showed loss of 5hmC expression in tumor cells as compared to normal smooth muscle or *FH* wildtype smooth muscle tumors. Loss of 5hmC in *SDH*-deficient tumors correlated significantly with nuclear exclusion of TET1 protein.

Our results agree with findings reported by Müller *et al*. [28], who demonstrated that nuclear exclusion of TET1 is associated with loss of 5hmC in gliomas. In contrast, *FH*-deficient smooth muscle tumors showed nuclear exclusion of TET1 in only 40% of cases and exclusion was not correlated with loss of 5hmC. Given that that oxidation of 5mC to 5hmC is considered to be a nuclear event, these results suggests TET1 is not the main player in the hydroxylation of 5mC in *FH*-deficient smooth muscle tumors. Thus far, little information is available on post-translational modifications of TET enzymes that may determine their subcellular localization. TET1 has three nuclear localization signals, suggesting a mainly nuclear localization of the protein. In addition, Xiao *et al.* [16] showed in HEK293 cells that stable knockdown of *SDHA/B* or *FH* reduced both TET1- and TET2-induced 5hmC levels as compared to control cells with normal *SDH* and *FH* expression. Therefore, in addition to TET1, the TET2 protein may also be associated with loss of 5hmC in *SDH*- and *FH*-deficient tumors, especially in *FH*-deficient HLRCC. Future studies should explore the role of TET2, principally in a much larger cohort of *FH*-deficient tumors than presently available. Also of note, we confirmed that 2-succinocysteine (2SC) immunohistochemistry is a robust biomarker for *FH* mutation status, consistent with earlier reports [24, 26]. In addition to the correct identification of two tumors from a HLRCC patient with a previously determined *FH* mutation, two sporadic LMS tumors were also positive for 2SC. However, insufficient paraffin-embedded tissue was available to allow *FH* germline mutation analysis and therefore the mutation might be somatic. Our results are consistent with earlier reports of very low *FH* mutation rates in LMS, including those of Kiuru *et al.* [31] who found germline *FH* mutations in 1-2% of apparently sporadic early-onset LMS, and Barker *et al.* [32], who observed no *FH* mutations in 26 sporadic LMS.

Besides the inhibition of the TET family of DNA hydroxylases, accumulation of succinate and fumarate negatively affects the enzyme activity of histone demethylases [14-16, 22]. We found an increased expression of H3K4me3 and H3K9me3 in chief cells compared to sustentacular cells in *SDH*-deficient tumors. However, we also found that H3K4me3 and H3K9me3 were highly expressed in the chief cell compartment of normal carotid bodies. Previous studies have used *SDH* wildtype PGL/PCC or adrenal glands with low expression levels of different histone methylation markers as a control group [11, 20]. This might explain differences with earlier results, since in our study chief cell expression levels of H3K4me3 and H3K9me3 are higher in *SDH*-deficient tumors compared to non-*SDH* mutant tumors, but did not differ significantly from the levels present in normal carotid bodies.

Regarding PCCs, we separated tumors into *VHL* mutant and non-mutant groups, as Letouzé *et al*. previously reported three stable DNA methylation clusters for PGLs and PCCs, including *SDH*x tumors, *VHL* tumors, and *NF1*-*RET*. The group differences in DNA and/or histone methylation suggested in this previous study were supported by our data, which showed increased H3K4me3 and H3K9me3 levels in *VHL* mutant PCC tumor cells compared to non- *VHL* mutant PCC. This finding lends support to the idea that changes in histone methylation may not be due to a direct effect of succinate accumulation, but may relate to the stabilization of HIF1, as HIF1 can directly regulate the activity of some JmjC domain-containing histone demethylases [33].

Of note, our study supports the concept of the chief cell compartment as the sole source of tumor cells in PGL, in agreement with previous reports [14, 34], and also underlines the curious heterogeneity of cells found in these tumors. Plotting sustentacular-chief cell ratios of H3K9me3 expression per tumor sample showed that expression levels of H3K9me3 were significantly increased in *SDH*-deficient tumors compared to non-*SDH* mutant tumors. This finding suggests that intra-tumor heterogeneity can mask differences in H3K9me3 levels. Furthermore, the involvement of SDH in modulating H3K9me3 levels was confirmed by *in vitro* silencing of *SDHB, SDHD* or *SDHAF2*, supporting the immunohistochemistry results in tumors. Despite small numbers, our results showed significantly elevated H3K9me3 levels in *FH*-deficient smooth muscle tumors, supporting the hypothesis that fumarate inhibits histone demethylation.

In contrast to H3K9me3, neither *SDH* nor *FH* mutant tumors displayed elevated H3K27me3 expression levels compared to control groups. These data contrast with other reports in which *SDH* mutant tumors reportedly showed an increased expression of H3K27me3 compared to *SDH* wildtype tumors [11]. A possible explanation for this difference might be the inclusion of predominantly *SDHB* mutated tumors in the earlier study, whereas our cohort consisted of mostly *SDHD* and *SDHAF2*-related tumors, which are known to have similar gene-expression profiles [35]. Letouzé *et al.* [11] reported a significantly higher mean level of hypermethylation in *SDHB*-mutated PGL/PCC compared to other *SDH* PGL/PCC, which might explain the difference in outcomes.

Overall, we found a similar pattern of epigenetic deregulation in *FH*-deficient smooth muscle tumors and *SDH*-deficient PGL/PCC, with loss of 5hmC proving a robust marker of deregulated DNA methylation. Like DNA methylation, H3K9me3 is often associated with regulatory elements of transcriptionally repressed genes and constitutive heterochromatic regions of the genome, and was also increased in both *SDH*- and *FH*-deficient tumors. Although not directly targetable, loss of SDH and FH do afford clinical opportunities such as synthetic lethal interactions. The DNA methyltransferase inhibitors 5-azacytidine and decitabine are of particular interest, where 5-azacytidine has been shown to reduce the proliferative index in an *in vivo* IDH1 glioma model [36] and decitabine repressed the migration capacities of Sdhb^−/−^ cells [11]. This could lead to clinical opportunities of epigenetic targeting in tumors caused by TCA cycle defects.

## MATERIALS AND METHODS

### Tissue samples

Formalin-fixed paraffin-embedded (FFPE) tissue samples of head and neck paragangliomas (PGL), pheochromocytomas (PCC), leiomyomas (LM) and leiomyosarcomas (LMS) were retrieved from the archives of the Department of Pathology. The histological appearances of all cases and controls were reviewed (JVMGB, JPB, PCWH, MAdG). For all PGL/PCC tumors and normal carotid bodies, the diagnosis was confirmed by routine S-100 immunohistochemical staining detecting sustentacular cells and chromogranin A detecting chief cells (Supplemental Figure 1A, 1B). In the smooth muscle tumors, at least one of the smooth muscle markers h-caldesmon or desmin was positive. PGL/PCC tumors were benign. The tissue samples were arrayed in tissue microarray (TMA) format resulting in a TMA including 100 PGL and 17 PCC samples in triplicate as previously described [37]. The TMAs were constructed using 0.5mm diameter punch (Beecher Instruments, Silver Spring, MD) to transfer tumor punches to the recipient block. Cores from human adrenal medulla, adrenal cortex, kidney, and liver were included for control and orientation purposes. TMAs with LM and LMS tumor samples were constructed from a panel of FFPE tumors including 7 uterine LM and 44 LMS as described previously [38]. Cores from colon, liver, placenta, prostate, skin, and tonsil were included for control and orientation purposes. As normal controls, we used whole sections of 6 normal carotid bodies, obtained from patients at autopsy within 24 hours after death, and 13 whole sections of normal smooth muscle of uterus, carotid artery, oesophagus, bowel wall, and aorta. In addition, we included whole sections from 9 *SDHAF2* PGL tumors obtained from Radboud UMC, Nijmegen, Netherlands, 8 *VHL* (Von Hippel-Lindau) PCC tumors obtained from Erasmus MC, Rotterdam, Netherlands, 2 uterine LM, and 2 cutaneous LM from two patients; 1 HLRCC patient with a germline *FH* mutation [39], and 1 suspected HLRCC patient (based on clinical data) with a *FH* mutation as detected by 2SC staining. The mutation status for most tumors was known (Table 1) and nonfamilial tumors with an unknown mutation status were excluded for analysis. Related patient clinical and genetic data for tumors included in the study is provided in supplemental Table 1. All samples were handled according to the Dutch code of proper secondary use of human material approved by the Dutch society of pathology (www.federa.org), and samples were handled in a coded (pseudonymised) fashion according to procedures agreed with the LUMC ethical board.

### Immunohistochemistry and scoring

The primary antibodies used in immunohistochemistry analysis are described in supplemental Table 2, with tonsil, colon and liver acting as positive controls. After antigen retrieval by microwave heating in Tris-EDTA buffer, pH 9.0 or citrate buffer, pH 6.0 at 100°C for 10 min, sections were blocked for 30 min with 10% goat serum and incubated overnight at 4°C with primary antibodies. Signal detection was performed with Envision+ (DAKO K3468, Agilent Technologies, Belgium) and the chromogen 3,3′-diaminobenzidine according to manufacturer's instructions.

The results of the immunohistochemical labeling were scored semi-quantitatively: the intensity of labeling was assessed on a scale of 0 to 3 (0 = none; 1 = weak; 2 = moderate; 3 = strong), and the percentage of positive cells was assessed on a scale of 0 to 4 (0 = 0% positive; 1 = 1-24% positive; 2 = 25-49% positive; 3 = 50-74%; 4 = 75-100% positive cells). The two scores were then added to find a total sum score ranging from 0-7, as described previously [40]. Chief cells and sustentacular cells were scored separately, if possible. For TET1, only subcellular localization was scored, as described [28]. In addition, TET1 expression was only scored in chief cells in PGL/PCC tumors.

A tumor was scored negative only when a positive internal control was present. Tumor samples were excluded from the analysis when substantial tissue was lost during sectioning. The scoring was performed independently by two observers blinded for clinicopathological data (ASH and JVMGB) and discrepancies were discussed. Immunohistochemistry images were taken using a Leica DFC550 camera with LAS software version 4.5 (Heerbrugg, Switzerland).

### Cell culture

HEK293 cells were obtained from DSMZ (ACC 305, Braunschweig, Germany) and grown in Dulbecco's Modified Eagle Medium (DMEM, Life Technologies, Paisley, UK) supplemented with 10% fetal bovine serum (Life Technologies) and penicillin/streptomycin (Life Technologies). HEK293 cells were maintained at 37°C in a humidified atmosphere of 5% CO2 in air.

### Lentiviral vector-based silencing of SDHD, SDHB and SDHAF2

To silence *SDHD, SDHB* and *SDHAF2*, three validated MISSION® shRNA constructs (TRCN0000231553 -236398, -159253 respectively) targeting human *SDHD* (NM_003002.1), *SDHB* (NM_003000.2), and *SDHAF2* (NM_017841.2) (Sigma Aldrich, St. Louis, USA) or scramble shRNA encoding plasmid (SHC002 Sigma Aldrich) were used to produce infectious virus particles (LV). To evaluate the transduction efficiency, the MISSION TurboGFP control plasmid (SHC003 Sigma Aldrich) was used. HEK293T cells were transfected with the shRNA constructs together with helper plasmids encoding HIV-1 gag-pol, HIV-1 rev, and the VSV-G envelope as described [41]. Viral supernatants were added to HEK293 cells in fresh medium supplemented with 8 μg/ml Polybrene (Sigma Aldrich) and the cells were incubated overnight. The next day, the medium was replaced with fresh medium. Transduction efficiency was analysed 3 to 6 days post transduction by evaluating GFP labelled cells. Experiments were performed 2-3 and 4-5 weeks after transduction of HEK293 cells with shRNAs.

### Western blotting

For preparation of total protein extracts, cells were extracted in RIPA buffer (Sigma Aldrich) supplemented with “complete” protease inhibitor cocktail (Roche, Germany). Total histone fractions were prepared using sodium dodecyl sulfate (sds) buffer containing 1% SDS, 10mM EDTA, and 10mM Tris pH 7.4, supplemented with “complete” protease inhibitor cocktail (Roche) and phosSTOP (Roche). The concentration of protein was determined by bicinchoninic acid protein assay (Thermo Scientific Pierce, Rockford, USA). Equal amounts of protein (30 μg) were separated by SDS-PAGE and transferred onto polyvinylidene fluoride (PVDF) membranes (Millipore). After blocking with 5% (w/v) non-fat milk powder, membranes were incubated overnight at 4°C with the following antibodies: SDHB 1:500 (Sigma Aldrich), α-tubulin 1:2000 (Sigma Aldrich), H3 and H3K9me3 1:2000 (Abcam, Cambridge, UK), H3K4me3 1:1000 and H3K27me3 1:2000 (Millipore, Billerica, USA). Visualization and quantification was carried out with the LI-COR Odyssey® scanner (Bad Homburg, Germany) and software (LI-COR Biosciences).

### Succinate and fumarate quantification by LC-MS/MS

Sample preparation for biochemical analysis of HEK293 cells with knockdown of *SDHD, SDHB* or *SDHAF2* and scrambled cells was performed according to [42], using ice cold 90% MeOH: CHCl_3_ as extraction solvent containing ^13^C-labeled isotopes of nucleotides as internal standards. Dried samples were reconstituted in 100μl H_2_O for compatibility with the liquid chromatography-tandem mass spectrometry (LC-MS/MS) method [43] and the concentrations of succinate and fumarate were determined by anion-exchange LC-MS/MS [44].

### Statistical analysis

IBM SPSS Statistics 20.0 for Windows software package (SPSS, Armonk, NY: IBM Corp) was used to analyze the results. The statistically significance of differences between 2 groups was assessed by the Mann-Whitney U test, and the 1-way analysis of variance test was used for comparisons of more than 2 groups. Statistical significance was determined by Pearson chi-square test to evaluate TET1 correlation with loss of 5hmC. *P* < 0.05 was considered statistically significant.

## SUPPLEMENTARY MATERIAL FIGURES AND TABLES




