# Supplementary material for: Inactivation of SDH and FH cause loss of 5hmC and increased H3K9me3 in paraganglioma/pheochromocytoma and smooth muscle tumors
Source: Oncotarget. 2015 Oct 12;6(36):38777–88. doi: 10.18632/oncotarget.6091 (PMC4770736; doi:10.18632/oncotarget.6091)
Supplement: Supplementary file 1 [file oncotarget-06-38777-s001.pdf]

Inactivation of *SDH* and *FH* cause loss of 5hmC and increased H3K9me3 in paraganglioma/pheochromocytoma and smooth muscle tumors

Supplementary Material

A **B**

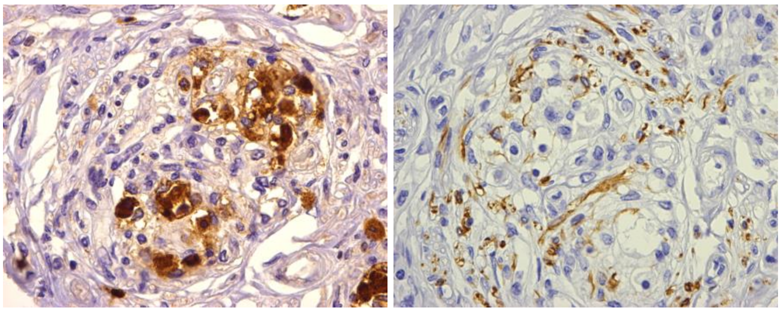

Figure S1. (A) Nuclear staining of chief cells with chromogranin A antibody in normal carotid body. (B) Nuclear and cytoplasmic staining of sustentacular cells in normal carotid body using an antibody directed to S100. Brown staining highlights the typical zellballen structures (40x magnification).

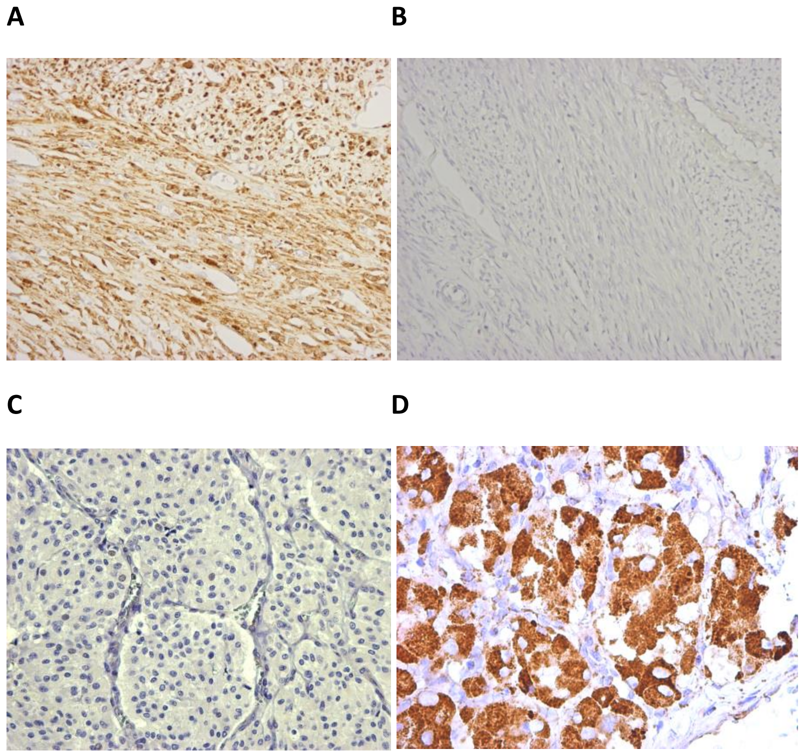

Figure S2. Immunohistochemical staining of tumors from (A) HLRCC patients expressing high levels of 2SC, which were undetectable in (B) *FH* wildtype LM/LMS tumors. (C) Negative SDHB staining in *SDHB, C, D and AF2* mutant tumors. (D) Positive SDHA staining in *SDHB, C, D and AF2* mutant tumors. Representative staining results are given (40x magnification).

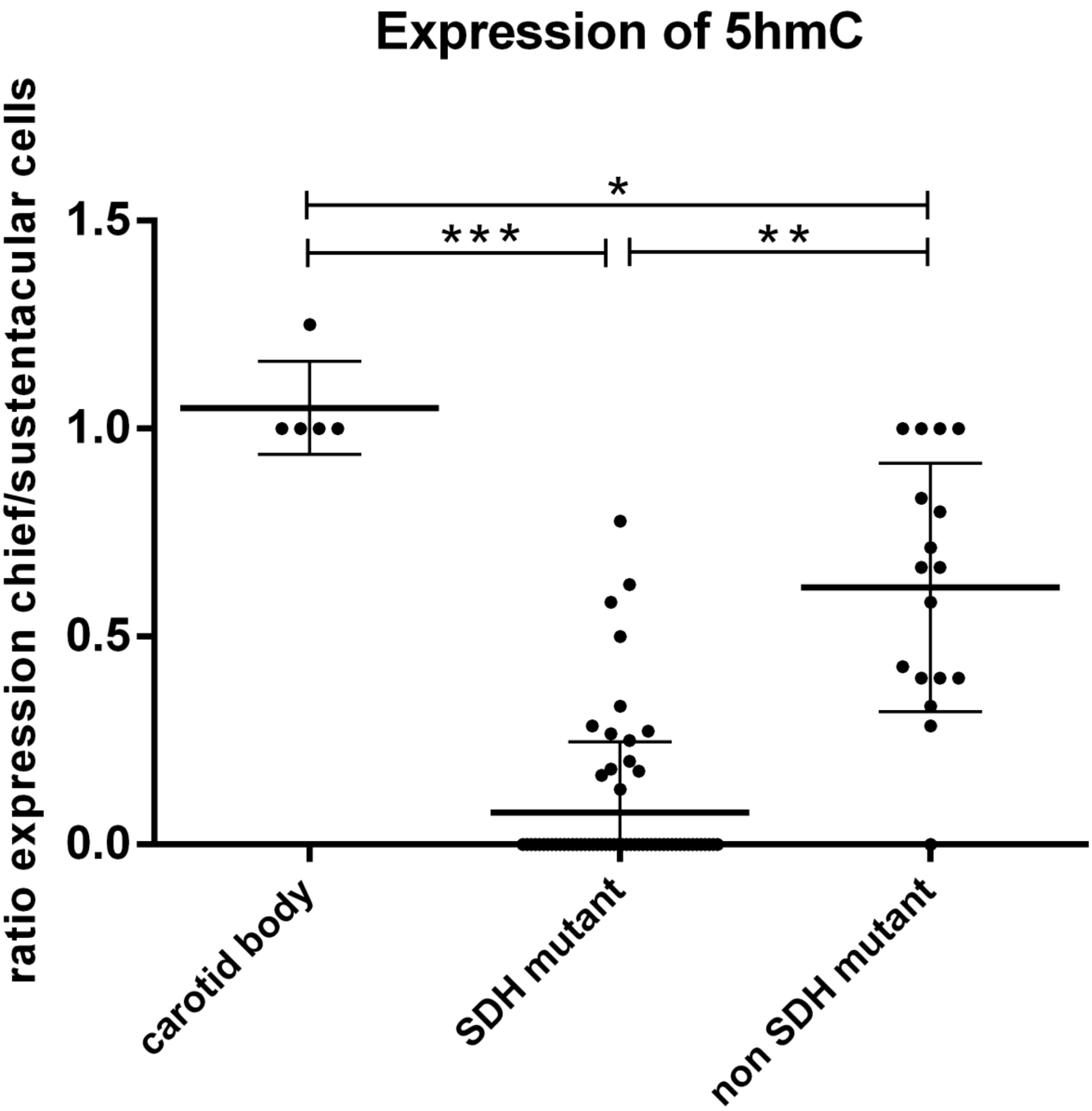

Figure S3. The ratio of 5hmC expression in the chief cells and the sustentacular cells per tumor sample differed significantly between SDH-deficient tumors, normal carotid bodies, and non-SDH mutant tumors.

Table S2. Primary antibodies used for immunohistochemistry analysis.

Primary antibody	Detect	Company	Clone	Dilution	Antigen retrieval buffer
Rabbit polyclonal	trimethyl-Histone H3, Lys 4	Millipore		1:7000	Tris-EDTA
Rabbit polyclonal	trimethyl-Histone H3, Lys 27	Millipore		1:7000	Tris-EDTA
Rabbit polyclonal	trimethyl-Histone H3, Lys 9	Abcam		1:2000	Tris-EDTA
Mouse monoclonal	5-methylcytosine	Millipore	33D3	1:2000	Tris-EDTA
Rabbit polyclonal	5-hydroxymethylcytosine	Active motif		1:7000	Citrate
Rabbit polyclonal	tet oncogene 1	GeneTex		1:800	Citrate
Rabbit polyclonal	2SC*	Eurogentec		1:1000	Citrate
Rabbit polyclonal	SDHB	Atlas		1:4000	Tris-EDTA
Mouse monoclonal	SDHA	Abcam	2E3GC12FB	1:2000	Tris-EDTA

*provided by Norma Frizzell
